# Refractory hypotension and coronary artery spasm induced by antipsychotic drugs: A challenging case and treatment consideration: A case report and literature review

**DOI:** 10.1097/MD.0000000000036400

**Published:** 2023-12-15

**Authors:** Kang Song, Yang Ji, Kun Zhao, Xiaorong Han, Chuanmin Jian, Song Liu

**Affiliations:** a Department of Cardiovascular Medicine, Qingdao University, Qingdao, China; b Department of Cardiovascular Medicine, The Affiliated Hospital of Qingdao University, Qingdao, China; c Department of Cardiovascular Medicine, Qingdao Central Hospital, Qingdao, China.

**Keywords:** antipsychotic drugs, coronary artery spasm, dobutamine, norepinephrine, refractory hypotension

## Abstract

**Rationale:**

Coronary artery spasms may result from supply–demand mismatch due to hypotension. Norepinephrine is more effective in ameliorating antipsychotic-induced refractory hypotension.

**Patient concerns:**

Postoperative difficult-to-correct hypoperfusion occurs in patients with comorbid depression and coronary spasm; the use of norepinephrine and epinephrine for rapidly raising blood pressure needs to be considered.

**Diagnoses:**

Electrocardiogram is an auxiliary tool and Digital Substraction Angiography is the gold standard for the diagnosis.

**Interventions:**

Surgery and correct choice of raising blood pressure are the main treatment methods.

**Outcomes:**

Hypotension induced by the use of antipsychotics after angiography is difficult to correct with dobutamine, and the above scenario is relatively rare in the clinic, where norepinephrine could be a potential therapeutic option.

**Lessons:**

Based on the lessons learnt from this case, caution must be exercised when dealing with patients on multiple antipsychotics during the perioperative period, while pressor-boosting medications should not be limited to conventional drugs such as dopamine. Norepinephrine may be more effective in dealing with difficult-to-correct hypoperfusion.

## 1. Introduction

Coronary heart disease has become one of the most serious diseases that take human life,^[1]^ coronary artery spasm is one of the coronary atherosclerotic heart disease. At the same time, with the development of modern society, the psychological pressure of human beings has been increasing, and more and more people suffer from mental illness, they take anti-psychotic drugs. Coronary artery spasms may result from supply–demand mismatch due to hypotension.^[2]^ When patients with coronary artery disease take antipsychotics, we need to pay attention to their drug interactions during clinical use so as to avoid underdiagnosis, misdiagnosis and delayed treatment. In this case, we report a case of persistent hypotension and coronary artery spasm after digital substraction angiography in a patient with coronary artery disease on antipsychotics, discussing its clinical features and drawing lessons from it, and caution should be exercised in the future in the use of a variety of antipsychotics in the perioperative period. The patient has provided informed consent for the publication of the case.

## 2. Case report

A patient was admitted to our urological department with a diagnosis of “double hydronephrosis for one month.” The patient had a history of hypertension for more than 20 years, with blood pressure up to 200/150 mm Hg. The patient was on amlodipine benzoate tablets (once daily) to maintain blood pressure control at 130/90 mm Hg. The patient has been taking quetiapine fumarate (0.1 g each night), magnesium valproate extended-release tablets (0.25 g 12 hourly), duloxetine hydrochloride enteric capsules (40 mg each night) for 6 years to manage depression. The patient had no history of diabetes mellitus, smoking, and alcohol consumption. The following tests were performed on admission: (1) blood tests: N-terminal B-type natriuretic peptide 84.36 pg/L (reference value 0–125 pg/L), creatine kinase isoenzyme 5.82 µg/L (reference value < 3.77 µg/L), myoglobin 90.25 µg/L (reference value 25–58 µg/L), and high-sensitivity troponin T 0.127 µg/L (reference value < 0.017 µg/L); (2) Imaging: coronary CT 3D imaging: coronary artery atherosclerosis with severe stenosis of the proximal segment of the anterior descending branch. Consequently, the patient was referred to our department for Digital Substraction Angiography. Based on the patient’s past medical history, symptoms and signs, and auxiliary examinations, the current diagnosis includes acute coronary syndrome, coronary atherosclerotic heart disease, heart failure (New York Heart Association class II), hypertension grade 3 (indicating a very high risk), neurogenic bladder, cerebral infarction, and depression.

The patient received preoperative routine anticoagulation and dual antiplatelet therapy after being transferred to our department. After excluding relevant contraindications, the patient underwent coronary angiography under X-ray cardiac monitoring on March 28, 2023. Intraoperatively, it was observed that the left anterior descending artery (LAD) had a 50% stenosis in the proximal segment (p-LAD), with thrombolysis in myocardial infarction flow grade of 3. The remaining vessels showed no abnormalities, and the patient had no discomfort during the operation and returned to the ward at 14:57. Three hours after the operation, the patient’s family expressed concerns about the patient’s appearance, noting paleness, profuse sweating, and cyanotic lips. Immediately assessment was conducted, and cardiac monitoring revealed the following: sinus rhythm, heart rate of 53 beats/min, respiratory rate of 18 breaths/min, blood pressure of 72/54 mm Hg, and oxygen saturation of 97%. Despite the administration of repeated atropine via intravenous push (1 mg), the patient’s blood pressure remained low at 57/41 mm Hg, with a heart rate of 96 beats per minute. In response, the patient received an intravenous drip of 5% dextrose sodium chloride (500 mL) under pressure, followed by an intravenous push of dobutamine (5 mg) and a continuous intravenous infusion of a mixture of 0.9% sodium chloride (50 mL) and dobutamine (200 mg). After 15 minutes of continuous pumping, the patient’s blood pressure did not increase significantly and was maintained at 67/45 mm Hg. Three hours and 30 minutes after the operation, the patient complained of anterior heart pain, and the electrocardiogram (ECG) showed sinus rhythm and exhibited significant ST-T dynamic changes in the anterior wall leads, as well as leads II, III, and aVF. The patient had a heart rate of 111 beats/min, respiratory rate of 17 breaths/min, blood pressure of 61/40 mm Hg, and oxygen saturation of 96%. The patient’s blood pressure was maintained at 75/37 mm Hg. The dobutamine infusion was discontinued, and norepinephrine (8 mg) was administered intravenously to regulate blood pressure. Resultantly, the patient’s blood pressure increased and was maintained at 100/68 mm Hg. The patient received Isosorbide mononitrate (30 mg) via intravenous micropump to improve coronary artery spasm. Five hours after the operation, the patient was quietly resting, and the cardiac monitor showed that the heart rate was 83 beats/min, respiratory rate was 18 breaths/min, blood pressure was 123/71 mm Hg, and oxygen saturation was 96%. The patient was given quetiapine fumarate (0.1 g), magnesium valproate extended-release tablets (0.25 g), and duloxetine hydrochloride enteric coated capsule (40 mg) after the operation to manage psychosis. The next day, the patient’s blood pressure was 116/68 mm Hg, and heart rate was 67 beats/min. The patient did not complain of discomfort and was discharged from the hospital. After discharge, the patient continued to take aspirin (100 mg once daily), resulvastatin (10 mg each night), amlodipine besylate (5 mg once daily), and nicorandil (5 mg 8 hourly). The patient has not experienced any further difficult-to-correct hypoperfusion and coronary microcirculation disorders while taking the antipsychotic drugs.

## 3. Discussion

Hypotension following coronary angiography can be caused by several complications, including extubation syndrome, vagal reflex, and pericardial tamponade. Extubation syndrome refers to a set of clinical symptoms, including bradycardia, low blood pressure, chest tightness, sweating, nausea, and vomiting, that some patients may experience when extubated after coronary angiography. Vagal reflex is generally a benign process and can be rapidly resolved with appropriate treatment. However, if not actively and promptly treated, the blood pressure and heart rate may become too low, with irreversible and serious consequences, or even death in patients with conditions such as severe valve disease or coronary artery disease. It is believed that the mechanism behind the vagal reflex involves various factors. Painful extraction, mental tension, fear, inadequate anesthesia, bleeding, compression hemostasis, and pressure bandaging can stimulate the nerves in the pubic area. Additionally, operation injury and other stimulating factors act on the cortical center and hypothalamus. These factors collectively lead to an abrupt increase in the tension of cholinergic vegetative nerves, triggering a strong reflex dilatation of small blood vessels in the viscera and muscles, resulting in a sharp drop in blood pressure and rapid slowing of heart rate. It can occur within 1 minute at the earliest. Pericardial tamponade refers to intraoperative or postoperative bleeding that leads to pericardial fluid accumulation. This accumulation restricts the ability of the pericardium to expand, resulting in compression of the heart and a decrease in cardiac ejection volume. Symptoms of pericardial tamponade typically include decreased blood pressure and tachycardia. In the case described, 3 hours after coronary angiography, the patient exhibited symptoms such as pallor, sweating, cyanotic lips, heart rate of 53 beats/min, respiratory rate of 18 breaths/min, and blood pressure of 72/54 mm Hg. While extubation syndrome and pericardial tamponade were not initially considered, the possibility of a vasovagal reflex could not be ruled out. Despite the repeated administration of intravenous atropine (1 mg), the patient’s hypotension remained difficult to correct. There have been reports of cases of quetiapine overdose leading to difficult-to-correct hypotension.^[3]^ While quetiapine overdose typically presents with hypotension and drowsiness, and in severe cases, coma, there have been no reported instances of quetiapine overdose directly causing coronary microvascular disease. In the case of our patient, the postoperative difficult-to-correct hypoperfusion and coronary artery spasm were associated with quetiapine. Quetiapine acts as an antagonist on various receptors, including dopaminergic (D2), 5-hydroxytryptaminergic (5-HT 2A, 5-HT 2C), histaminergic (H1), muscarinic (M1, M3), and adrenergic (α1, α2) receptors. Several hypotheses have been proposed to explain the binding of quetiapine to these receptors. From these hypotheses, it can be concluded that quetiapine exerts an antagonistic effect on adrenergic α1 receptors and that upregulation of this receptor in the brainstem induces both bradycardia and hypotension. This conclusion is consistent with the hypotension observed in our patient, who exhibited an inadequate response to dobutamine on multiple occasions. Following the administration of norepinephrine, the patient’s blood pressure gradually increased and stabilized at 110/60 mm Hg. Considering the mechanism of action of norepinephrine,^[4]^ we understand that norepinephrine has relatively limited activity on β2-adrenergic receptors, which are responsible for relaxing vascular smooth muscle and reducing peripheral resistance. Thus, norepinephrine increases peripheral resistance, and compensatory vagal reflexes tend to overcome its positive chronotropic effects on the myocardium while preserving positive inotropy. Considering the pharmacological profile of quetiapine (especially its α1 antagonism), the α1 vasoconstrictor effect of norepinephrine, which is independent of the vasodilating β2 effect, theoretically makes it the most suitable drug for reversing hypotension after quetiapine overdose.

In addition to the difficult-to-correct hypotension, the patient in this case also exhibited significant ST-T dynamic changes in the anterior wall leads, as well as leads II, III, and aVF. However, the coronary angiography results showed non-obstructive lesions, normal vasodilation, and thrombolysis in myocardial infarction flow grade 3. A 50% stenosis was observed in the p-LAD, while the remaining vessels did not exhibit significant stenosis. After the patient started taking antipsychotic drugs postoperatively, the phenomenon of difficult-to-correct hypoperfusion occurred. The patient then complained of chest pain, and the ECG showed ST-T depression in the anterior chest leads, which indicated significant changes compared to the previous readings. These findings, in light of the patient’s substantial deficit in myocardial vascular perfusion, suggest the occurrence of myocardial ischemia. The coronary microvessels were likely functionally and structurally damaged, resulting in reduced blood flow and dysfunction in vasodilation.^[5]^ The degree of ischemia caused by coronary artery spasm is not fixed, and therefore the ECG changes may include not only ST-segment elevation but also ST-segment depression or T-wave changes, as evidenced by the ECG before and after the onset of chest pain in this patient. We reviewed relevant literature and found that myocardial ischemia can occur in more than 1/3 of patients with coronary artery disease during psychological stress. This type of ischemia is called psychological stress myocardial ischemia and occurs mostly in postmenopausal women.^[6]^ It is known to have both physical and psychological triggers. In this particular case, the patient’s symptoms were significantly relieved upon discontinuation of the antipsychotic drugs, and neither hypotension nor coronary artery spasm recurred. However, current literature does not provide evidence suggesting that antipsychotics can cause or exacerbate coronary spasms, and further studies are needed.

The present study has some limitations: (1) The case study is not representative of the response to antipsychotics in the population, and experimental studies with large samples are needed to provide evidence-based medical evidence. (2) The present study is limited by ethnicity and only represents the response of older Asian women, and multicenter studies are needed to further clarify the mechanisms by which antipsychotics produce uncorrectable hypoperfusion that leads to coronary artery spasm.

## 4. Conclusion

In conclusion, when prescribing antipsychotics to patients at risk of cardiac morbidity, it is crucial to consider the potential interactions and risks associated with these psychotropic drugs and their cardiac effects so that the most appropriate psychotropic drug can be selected for each patient. Regular monitoring of blood pressure, heart rate, and ECG is recommended to promptly detect any cardiac-related adverse effects and ensure patient safety.^[7]^ This case suggests that if postoperative difficult-to-correct hypoperfusion occurs in patients with comorbid depression and coronary spasm, treatment must not be limited to commonly used methods of raising blood pressure, such as atropine, dobutamine, and saline. Considering that there is mutual resistance between quetiapine and the above mentioned emergency medication, and that norepinephrine is an alpha agonist that achieves rapid vasoconstriction, contributing to higher blood pressure and increased coronary blood flow, contributing to myocardial and vasoconstriction. So the use of norepinephrine and epinephrine for rapidly raising blood pressure needs to be considered. The occurrence of difficult-to-correct hypoperfusion in depressed patients or myocardial ischemia in depressed patients is not uncommon in clinical practice. However, the coexistence of both symptoms in depressed patients is currently uncommon, and further exploration of the underlying causes is necessary. In summary, severe hypotension that does not respond to dobutamine is a relatively rare complication of quetiapine toxicity. However, when it occurs, the case characteristics and the pharmacodynamics of quetiapine suggest norepinephrine can be considered as a potential treatment option. Furthermore, caution must be exercised when using multiple antipsychotics in the perioperative period based on the lessons learned from this case.^[8]^

## Author contributions

**Data curation:** Xiaorong Han, Chuanmin Jian.

**Formal analysis:** Yang Ji, Kun Zhao.

**Supervision:** Song Liu.

**Writing – original draft:** Kang Song.

**Writing – review & editing:** Song Liu.
